# Supplementary material for: Cone-driven, geniculo-cortical responses in canine models of outer retinal disease
Source: bioRxiv. 2023 Dec 14:2023.12.13.571523. Preprint. [Version 1] doi: 10.1101/2023.12.13.571523 (PMC10760074; doi:10.1101/2023.12.13.571523)
Supplement: Supplement 1 [file NIHPP2023.12.13.571523v1-supplement-1.pdf]

bioRxiv preprint doi: https://doi.org/10.1101/2023.12.13.571523; this version posted December 14, 2023. The copyright holder for this preprint (which was not certified by peer review) is the author/funder, who has granted bioRxiv a license to display the preprint in perpetuity. It is made available under aCC-BY 4.0 International license.

Cone-driven, geniculo-cortical responses in canine models of outer retinal disease

Supplementary materials

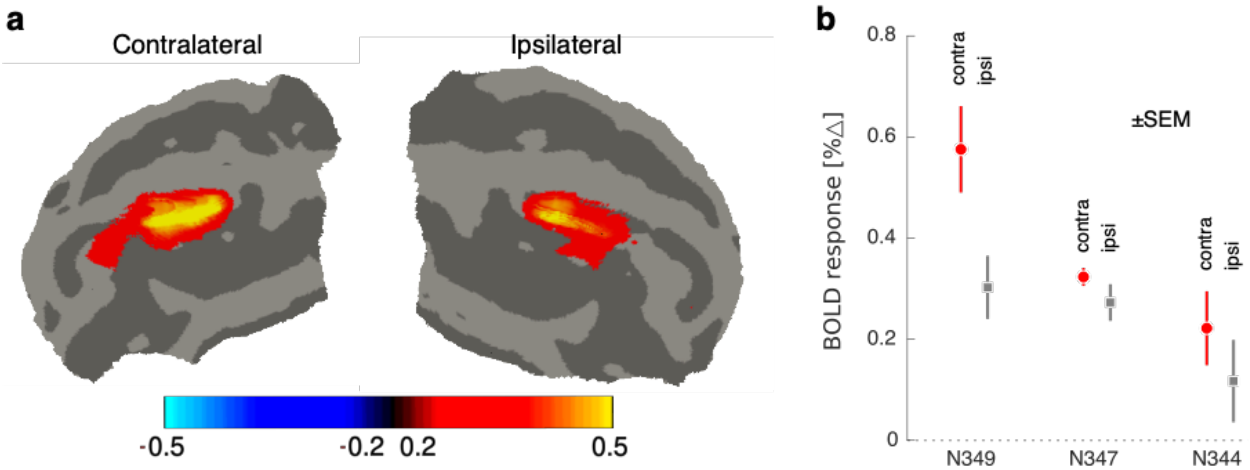

Figure S1: Visual cortex responses contralateral and ipsilateral to the stimulated eye. Data taken from WT animals and the light-flux stimulus. a) Surface maps showing the mean (across animals) percentage signal change to stimulation from the contralateral and ipsilateral eye. The amplitude and extent of response is slightly greater for contralateral stimulation. b) The response for each animal shows a consistent difference between the two eyes. Error bars are the standard error across acquisitions.

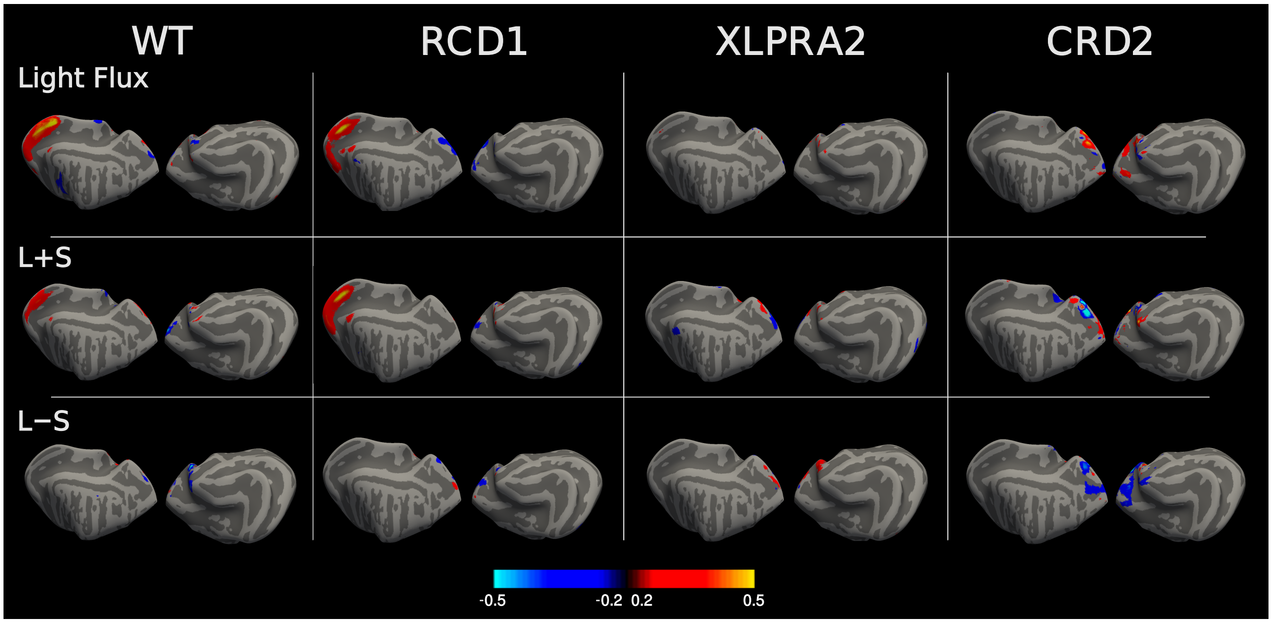

Figure S2: Whole brain maps of fMRI response; corresponds to Figure 5.

bioRxiv preprint doi: https://doi.org/10.1101/2023.12.13.571523; this version posted December 14, 2023. The copyright holder for this preprint (which was not certified by peer review) is the author/funder, who has granted bioRxiv a license to display the preprint in perpetuity. It is made available under aCC-BY 4.0 International license.

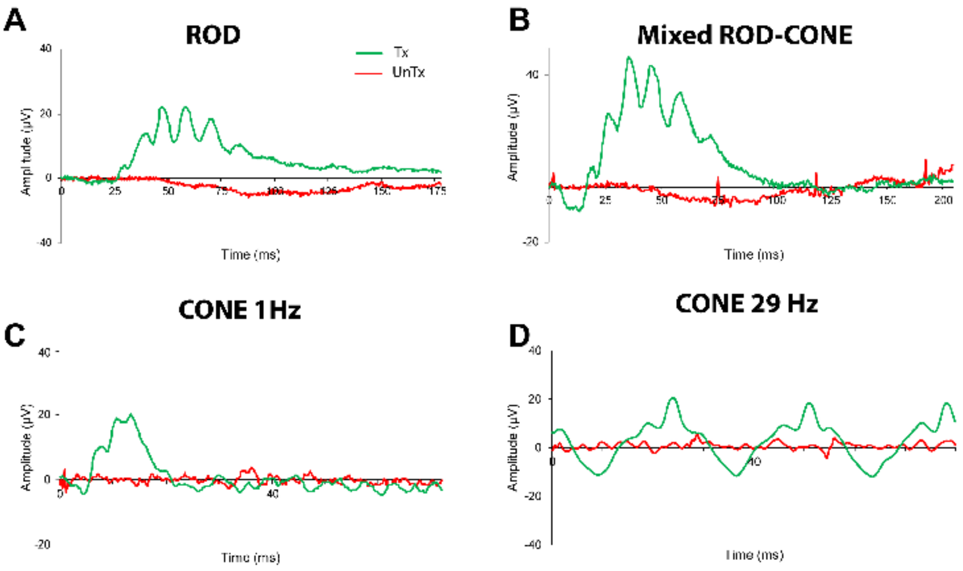

Figure S3: Electroretinographic recordings in a CRD2/*NPHP5* affected dog (WM67; age 38 weeks) at 24 weeks post-delivery of AAV2/5-NPHP5 to the treated (Tx, green traces) left eye. The contralateral right eye was untreated (UnTx, red traces)

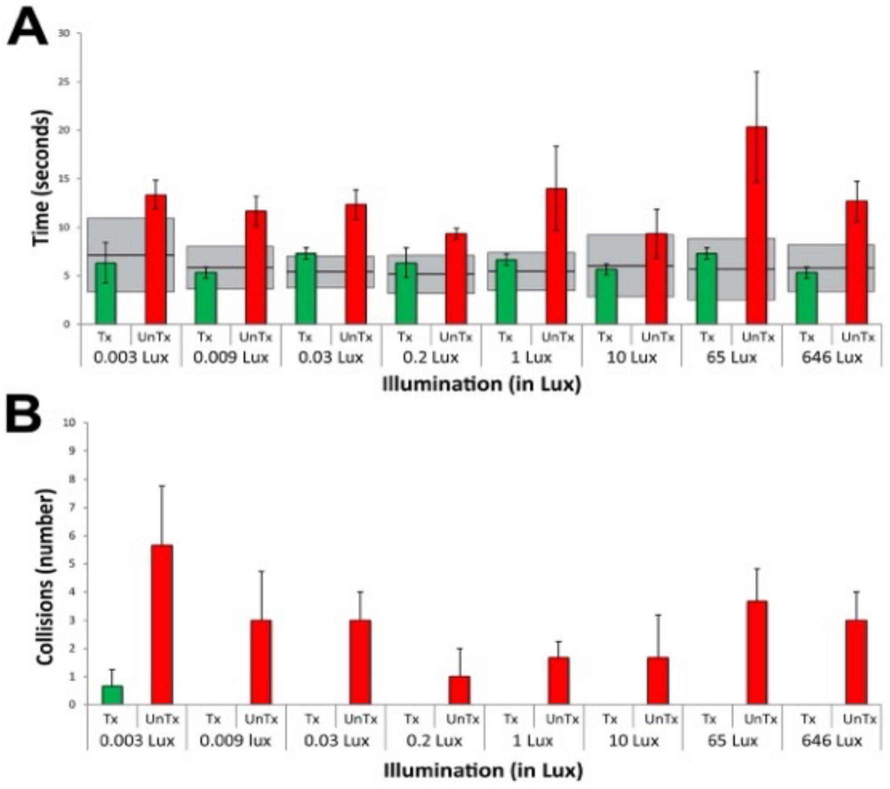

Figure S4: Visual function in an obstacle avoidance course under different ambient light intensities of a CRD2/*NPHP5* affected dog (WM67; age 38 weeks) at 24 weeks post-delivery of AAV2/5-NPHP5 to the treated (Tx, green bars) left eye. The contralateral right eye was untreated (UnTx, red bars) (**A**) Mean (±SD) transit time. Gray bars show 95% CI of normal (untreated) dogs. (**B**) mean (±SD) number of collisions (WT dogs have zero collisions).

bioRxiv preprint doi: https://doi.org/10.1101/2023.12.13.571523; this version posted December 14, 2023. The copyright holder for this preprint (which was not certified by peer review) is the author/funder, who has granted bioRxiv a license to display the preprint in perpetuity. It is made available under aCC-BY 4.0 International license.

Disease	Retinal function (ffERG)		Visual behavior (obstacle avoidance course)	
	Rod-mediated	Cone-mediated	Scotopic conditions	Photopic conditions
RCD1	Absent as early as 24 days[a]	Persistent at 18 months[b] and > 6 years[c]	Absent as early as 6-8 weeks[d]	Persistent for > 6 years[c]
XLPRA2	Nearly absent by 5 months[e]	Persistent at 24 months[f,g]	Absent by 27 months[c]	Persistent for > 6 years[c]
CRD2	Absent after 14 weeks[h]	Absent as early as 6 weeks[h]	Absent at 8 months[i]	Absent at 8 months[i]

Table S1: Summary of ages at which retinal and visual function are detectable in RCD1/*PDE6B*, XLPRA2/*RPGR*, and CRD2/*NPHP5* affected dogs.

ffERG: full field ERG

[a]: Aguirre GD, Rubin LF. Rod-cone dysplasia (progressive retinal atrophy) in Irish setters. *Journal American Veterinary Association*, 1975; 166 (2): 157-164.

[b]: Petit L, Lheriteau E, Weber M, et al. Restoration of vision in the pde6beta-deficient dog, a large animal model of rod-cone dystrophy. *Molecular Therapy*, 2012: 20 (11): 2019-2030

[c]: Beltran WA (personal communication)

[d]: Hodgman SFJ, Parry HB, Rasbridge WJ, Steel JD. Progressive Retinal Atrophy in dogs 1. The disease in Irish Setters (Red). *Veterinary Record*, 1949; 61 (15): 185-189.

[e]: Dufour VL, Cideciyan AV, Ye G-J, et al. Toxicity and efficacy evaluation of an adeno-associated virus vector expressing codon-optimized RPGR delivered by subretinal injection in a canine model of X-linked Retinitis Pigmentosa. *Human Gene Therapy*, 2020: 31 (3-4): 253-267.

[f]: Beltran WA, Cideciyan AV, Iwabe S. Successful arrest of photoreceptor and vision loss expands the therapeutic window of retinal gene therapy to later stages of disease. *Proceedings of the National Academy of Sciences of the USA*, 112 (43): E5844-5853.

[g]: Beltran WA, Cideciyan AV, Boye SE, et al Optimization of retinal gene therapy for X-linked retinitis pigmentosa due to RPGR mutations. *Molecular Therapy*, 2017; 25 (8): 1866-1880

[h]: Downs LM, Scott EM, Cideciyan AV et al. Overlap of abnormal photoreceptor development and progressive degeneration in Leber congenital amaurosis caused by NPHP5 mutation. Human Molecular Genetics, 2016; 25 (19): 4211-4226

[i]: Aguirre GD, Cideciyan AV, Dufour VL et al. Gene therapy reforms photoreceptor structure and restores vision in NPHP5-associated Leber congenital amaurosis. *Molecular Therapy*, 2021; 29 (12): 2456-2468

	Modulation	L+S	L-S	Mel	Rod
**MRI**	LightFlux	95.4± 0.8	-0.3 ± 0.3	95.2 ± 0.9	95.2 ± 0.9
	L-S	0.4 ± 2.2	25.2 ± 0.9	0.2 ± 0.5	0.3 ± 0.8
	L+S	35.5 ± 1.0	0.2 ± 1.3	-0.3 ± 1.1	0.4 ± 1.1
	Rod/Mel	-0.1 ± 1.0	-0.9 ± 2.5	50.8 ± 1.6	49.7 ± 1.2
**Pupillometry**	LightFlux	96.4 ± 0.9	0.1 ± 0.4	96.3 ± 0.9	96.2 ± 0.9
	L-S	-2.2 ± 2.5	26.0 ± 0.7	-0.3 ± 0.5	-0.5 ± 0.9
	L+S	34.9 ± 0.8	-1.4 ± 1.5	1.2 ± 1.3	-0.7 ± 1.1
	Rod/Mel	0.6 ± 0.4	2.5 ± 3.0	49.5 ± 2.0	51.7 ± 1.2

Table S2: Spectroradiometric measurements of the stimuli were made after each experimental session. Inevitable imprecision in device control leads to variation in contrast on the targeted photoreceptor populations. Shown is the mean and standard deviation across animals of the calculated contrast upon the targeted and silenced photoreceptor classes for the MRI and pupillometry studies.
